# Reframing Substance Misuse Prevention: a RE-AIM Analysis of Federal Infrastructure and Future Directions

**DOI:** 10.1007/s11121-026-01920-4

**Published:** 2026-06-30

**Authors:** Dane Minnick, Laura Curran, Khary K. Rigg

**Affiliations:** 1 Evelyn Duvall Chair of Family and Community Health, School of Social Work University of South Florida, Sarasota–Manatee Campus, Sarasota, FL USA; 2 Department of Behavioral Health Sciences and Practice, University of South Florida, Sarasota–Manatee Campus, Sarasota, FL USA; 3 Department of Behavioral Health Sciences and Practice, University of South Florida, Tampa, FL USA

**Keywords:** Prevention, Substance misuse, Policy, Digital health, Public health infrastructure

## Abstract

Substance misuse prevention is a significant component of public health policy in the USA. Currently, federal prevention efforts rely primarily on a coalition-based framework supported through four mechanisms: (1) the Substance Use Prevention, Treatment, and Recovery Block Grant, (2) the Drug-Free Communities Program, (3) the Partnerships for Success initiative, and (4) Community Anti-Drug Coalitions of America. Using the Reach, Effectiveness, Adoption, Implementation, and Maintenance (RE-AIM) framework, this article critically analyzes the performance of this system and considers scalable alternatives. Findings indicate that while coalitions mobilize local stakeholders, the model is resource-intensive, inconsistently implemented, and limited in its capacity to achieve sustained, population-level impact. Proposed alternatives include integrating prevention science into professional education, mandating EBPP delivery in universal settings such as schools, embedding routine screening and early intervention, creating a national repository of free prevention programs, and expanding the use of digital applications and wearable technologies. Implications include restructuring federal funding priorities, embedding prevention within existing infrastructures, and advancing research on cost-effectiveness, sustainability, and equity.

Substance misuse and addiction are major public health challenges in the USA that affect millions of people each year. According to the 2023 National Survey on Drug Use and Health (NSDUH), 47.5% of the population (134.7 million individuals) reported past-month alcohol use, 15.4% (43.6 million) reported marijuana use, and 22.7% (64.4 million) reported tobacco or nicotine vaping. An estimated 24.9% (70 million) used illicit drugs in the past year, and 48.7 million people aged 12 or older, or 17.3% of the U.S. population, met the criteria for a substance use disorder (Substance Abuse and Mental Health Services Administration (SAMHSA), 2024a). Findings from the 2023 Youth Risk Behavior Survey also underscore the impact of substance use on youth, with 22% of high school students reporting current alcohol use and 17% reporting marijuana use in the past 30 days (Centers for Disease Control and Prevention (CDC), 2024).

In an effort to prevent substance misuse beyond traditional enforcement and interdiction activities, federal and state governments have strongly invested in the development of a national prevention infrastructure centered on local community coalitions. The primary components of this system are the prevention allocation of the Substance Use Prevention, Treatment, and Recovery Block Grant (SUPTR-BG), the Drug-Free Communities (DFC) program, the Partnerships for Success (PFS) initiative, and Community Anti-Drug Coalitions of America (CADCA) (CADCA, n.d.; SAMHSA, 2019; 2023a; 2024b). Collectively, these mechanisms, in conjunction with technical assistance providers such as the Strategic Prevention Technical Assistance Center and the Prevention Technology Transfer Centers, position community-based coalitions as the primary vehicle for implementing prevention strategies and programs across the country.

While the coalition approach was historically designed to promote local ownership and allow for targeted interventions within the environmental contexts of the late 1900s, evidence suggests that coalition-based models face challenges regarding reach, implementation costs, impact, sustainability, and cost-effectiveness (Butterfoss & Kegler, 2002; Hallfors et al., 2002; Hutchison & Russell, 2021). Moreover, advances in prevention science, technology, and shifts in social norms have created new opportunities for delivering cost-effective, evidence-based prevention strategies at scale. These developments call into question the utility and return on investment of the current prevention infrastructure, which warrant further investigation.

To explore this issue, this article presents a critical analysis of the current coalition-based prevention system and considers alternative strategies using the Reach, Effectiveness, Adoption, Implementation, Maintenance (RE-AIM) framework. The RE-AIM tool is a widely used model for evaluating public health interventions, emphasizing both outcomes and real-world application (Glasgow et al., 1999, 2019). Specifically, the analysis considers: (1) system reach, (2) associations with substance misuse trends, (3) adoption and implementation of evidence-based prevention programs (EBPPs), (4) implementation demands, and (5) costs, return on investment, and long-term sustainability. The analysis performed is conceptual and policy-oriented in nature. It does not constitute a formal systematic review or causal evaluation of federal programs, but rather applies the RE-AIM framework to synthesize representative empirical evidence relevant to system-level performance. Implications for policy, practice, and future research are discussed.

## Background

### Prevention Infrastructure

The contemporary U.S. prevention system was first established in 1973 with the creation of the Alcohol, Drug Abuse, and Mental Health Administration (ADAMHA). ADAMHA originally provided federal funding to support state substance use and mental health services and served as the parent organization for the National Institute of Mental Health, National Institute on Drug Abuse, and National Institute on Alcohol Abuse and Alcoholism (Duff, 2020; United States Congress, 1992). Distinct from traditional interdiction and enforcement initiatives, these early efforts emphasized research, technical assistance and training, and funding support for states, which historically led individual efforts to address local behavioral health issues (Duff, 2020).

In 1992, Congress passed the ADAMHA Reorganization Act (Pub. L. 102–321), which transferred the research institutes to the National Institutes of Health and established the Substance Abuse and Mental Health Services Administration (SAMHSA) to oversee service-related substance use and mental health programs. This restructuring also created two distinct block grants: the Substance Abuse Prevention and Treatment Block Grant and the Community Mental Health Services Block Grant (Duff, 2020; United States Congress, 1992).

In 2021, SAMHSA informally updated the Substance Abuse Prevention and Treatment Block Grant’s name to the Substance Use Prevention, Treatment, and Recovery Services Block Grant (SUPTRS-BG) to better reflect contemporary terminology and to recognize the inclusion of recovery services. Under federal statute, states must allocate at least 20% of SUPTRS-BG funding to primary prevention activities. In fiscal year 2023, the block grant totaled $2.008 billion, with approximately $401.6 million designated for prevention (National Association of State Alcohol and Drug Abuse Directors [NASADAD], 2023; Duff, 2020; SAMHSA, 2023b). Beyond SUPTR-BG, SAMHSA maintains a dedicated prevention budget for administration, training, and technical assistance, overseen by the Center for Substance Abuse Prevention (CSAP). CSAP is tasked with developing federal prevention initiatives and providing oversight of contracted providers, public education, and the dissemination of research findings (Duff, 2020).

Several other federal initiatives also currently shape the prevention system. The Drug-Free Communities Support Program, which was established in 1997 through the Drug-Free Communities Act, serves as a central organization within the federal prevention system (United States Congress, 1997; CDC, 2025). Jointly administered and overseen by the Office of National Drug Control Policy, the CDC, and SAMHSA, DFC receives around $99 million per year in federal funding (2018–2023) to provide community coalitions with training and technical assistance, and to provide direct funding to coalitions to strengthen local prevention capacity and programming (Kane, 2021). The DFC program emphasizes youth prevention through multi-sector partnerships, including schools, law enforcement, health care providers, parents, and faith-based organizations. In 2024, there were 753 DFC coalitions operating nationwide and an additional 64 coalitions were funded through the Comprehensive Addiction and Recovery Act (CARA). CARA grants are awarded to coalitions previously funded through DFC to emphasize strategies specific to opioids, methamphetamines, and prescription drug misuse among youth ages 12–18 in communities with higher-than-average rates of use (CDC, 2025).

Alongside DFC, the Partnerships for Success program was launched by SAMHSA in 2006 to help states and communities address emerging substance use trends and to support populations with an elevated risk (SAMHSA, 2024b). With a proposed federal budget of $134.8 million for fiscal year 2025, PFS provides competitive grants to states, tribes, and jurisdictions to implement and evaluate evidence-based prevention interventions, with a frequent focus on underage drinking, prescription drug misuse, and other high-priority issues (NASADAD, 2025). Unlike DFC, which funds local coalitions directly, PFS operates primarily through state agencies that in turn sub-grant to local communities. In 2024, 38 states received PFS funding, supporting 177 community grantees and 18 tribal grantees (SAMHSA, 2024b).

In addition to DFC and PFS, Community Anti-Drug Coalitions of America plays a leading role in supporting the national prevention infrastructure. Founded in 1992 as a nonprofit membership organization, CADCA provides training, technical assistance, and pass-through funding for community coalitions across the U.S. With $16.4 million of revenue available via a multitude of federal contracts for fiscal year 2024, CADCA’s primary efforts have been composed of developing new prevention resources and facilitating training events such as the Mid-Year Training Institute and National Leadership Forum (USAspending, n.d.; CADCA, n.d.).

Lastly, the federal government has made additional investments into technical assistance and training efforts for the prevention workforce through the Strategic Prevention Technical Assistance Center (SPTAC) and the Prevention Technology Transfer Centers (PTTCs). SPTAC was established in 2022 to build prevention capacity specifically for SAMHSA grantees through training, consultation, and evaluation support (SAMHSA, 2024c). Complementing SPTAC are the traditional PTTCs, a national training and technical assistance system created in 2018 to encourage the dissemination and uptake of evidence-based prevention practices (PTTC Network, n.d.). SPTAC receives an estimated $5.5 million per year, while the PTTCs received $8.134 million in fiscal year 2024 to administer national prevention support services (HigherGov, 2023; SAMHSA, 2024d). A full breakdown of the national prevention system and annual funding estimates is provided in Table 1.

**Table 1:** Prevention infrastructure

Program	Founding	Purpose	Oversight	Admin	Annual federal funding (millions)
SUPTR‑BG	1992	Prevention infrastructure and programs	SAMHSA (CSAP)	State-level block grants	$401.6* (2023)
DFC	1997	Community coalitions	ONDCP and SAMHSA	Federal grants	$99.0 (2018–2023)
PFS	2012	Prevention services	SAMHSA	Federal grants	$134.8 (2025)
CADCA	1992	Coalition training/TA, grants, support	ONDCP	Nonprofit 501(C)(3)	$16.4 (2024)
SPTAC	2022	Grantee training/TA	SAMHSA	Contracted provider	$5.5 (2022–2027)
PTTCs	2018	Workforce training/TA	SAMHSA	Contracted centers	$8.1 (2024)
Total					$665.4

*Refers to the prevention allocation of SUPTR-BG

*SUPTR-BG—Substance Use Prevention Treatment and Recovery Block Grant

*DFC—Drug-Free Communities

*PFS—Partnership for Success

*CADCA—Community Anti-Drug Coalitions of America

*SPTAC—Strategic Prevention Technical Assistance Center

*PTTC—Prevention Technology Transfer Center

### Community Coalition Framework

Community prevention coalitions are multi-sector partnerships that bring together stakeholders such as schools, law enforcement, healthcare providers, community organizations, and neighborhood volunteers to plan and implement prevention assessments, programs, and strategies (CADCA, n.d.; SAMHSA, 2019). Supported by multiple funding sources, agencies, and technical assistance providers at an annual estimated cost of $665.4 million, community coalitions are a primary vehicle for implementing prevention policies and programs across the US.

The contemporary coalition framework is based on SAMHSA’s Strategic Prevention Framework (SPF), which was first introduced in 2004 as a guiding model for facilitating community prevention services (SAMHSA, 2019). In alignment with the SPF, coalitions are expected to follow a structured set of steps: assessment, capacity building, planning, implementation, and evaluation, with emphasis on both cultural competence and sustainability. In addition, all community coalitions funded through SUPTRS-BG are further expected to implement interventions in alignment with the six federally-approved prevention strategy categories: environmental strategies (e.g., crafting or influencing local laws and policies), community-based processes (e.g., stakeholder engagement and coalition development), information dissemination (e.g., awareness campaigns, social marketing), education (evidence-based curricula and programs), alternative activities (drug-free recreational and social events), and problem identification and referral (e.g., screening, referral, and early intervention) (CADCA, n.d.; SAMHSA, 2019; SAMHSA, 2024c).

## Methodology

This analysis applied the RE-AIM framework (Reach, Effectiveness, Adoption, Implementation, Maintenance) to evaluate the U.S. prevention system. Originally developed to improve translation of health research into practice, RE-AIM has been widely used to assess outcomes and feasibility across public health and clinical settings and has been cited in over 2,800 publications (Glasgow et al., 2019). Within the framework: Reach refers to the number, proportion, and representativeness of individuals who participate in a program or are exposed to a strategy; Effectiveness refers to the impact of an intervention on important outcomes, including both intended benefits and potential unintended consequences; Adoption concerns the number, proportion, and representativeness of settings and delivery agents that are willing to initiate a program; Implementation addresses the consistency, fidelity, and cost of delivering a program as intended in real-world conditions; and Maintenance refers to the extent to which programs or policies are sustained over time at both the individual and organizational levels (Glasgow et al., 1999, 2019).

Although RE-AIM was originally developed to evaluate individual health promotion interventions, its application has expanded substantially over the past two decades. Reviews of RE-AIM use demonstrate increasing application to dissemination and implementation research, multi-component strategies, and organizational and systems-level public health initiatives (Gaglio et al., 2013; Glasgow et al., 2019; Jilcot et al., 2007; Kessler et al., 2012). In addition, the community coalition framework evaluated here represents a structured, federally supported prevention delivery model with defined processes, fidelity expectations, and sustainability mechanisms under the Strategic Prevention Framework (CADCA, 2018; SAMHSA, 2019). As such, coalitions function not merely as passive infrastructure, but also as a federally-defined intervention strategy deployed across communities (CADCA, 2018; SAMHSA, 2019). Applying RE-AIM at the systems level is therefore consistent with contemporary implementation science and prior macro-level applications of the framework. Additional information on RE-AIM, as well as resources on how to utilize the framework can be found at re-aim.org.

In the presented analyses, the framework was not applied to a single intervention or individual coalition, but to both the coalition model and the coalition-based system as a whole, enabling comparison with proposed alternative strategies and funding priorities. Each RE-AIM domain was assessed using publicly available data and peer-reviewed research. Reach and Effectiveness were analyzed using population-level data from the NSDUH (2002–2019) and Monitoring the Future (MTF, 1992–2019). Thirty-day prevalence of alcohol, marijuana, nicotine, and illicit drug use served as proxy measures, supplemented by overdose mortality data and national economic cost estimates.

For Adoption, Implementation, and Maintenance, peer-reviewed studies were reviewed that assessed coalition impact, functioning, and sustainability, including evaluations of Communities That Care, the Robert Wood Johnson Foundation’s Fighting Back initiative, and other large-scale coalition assessments (e.g., Feinberg et al., 2008; Gloppen et al., 2012; Hallfors et al., 2002; Kreuter et al., 2000). Articles were identified using Google Scholar and Elicit, an AI-assisted literature review platform. Elicit operates by leveraging large language models to interpret research questions, retrieve candidate articles (primarily via Semantic Scholar), and extract key study details into structured tables (Elicit, 2025). Of note, Elicit has been found to demonstrate lower sensitivity than traditional database searching, although it can achieve higher precision in certain contexts. Current evidence suggests that Elicit should serve as a supplementary tool alongside conventional methods (Lau & Golder, 2025). Two prompts guided the Elicit search:

1. “What is the effectiveness and return on investment of community prevention coalitions funded through Partnerships for Success (PFS), Community Anti-Drug Coalitions of America (CADCA), and the Drug-Free Communities (DFC) program, including their implementation challenges, resource intensity, costs, and documented strengths and weaknesses? and
2. What is the reach, effectiveness, adoption, implementation, maintenance, and sustainability of community substance misuse prevention coalitions in the USA?

For Google Scholar, search terms included “effectiveness of community coalitions,” “effectiveness of substance misuse prevention coalitions,” “Partnerships for Success evaluation,” “Community Anti-Drug Coalitions of America evaluation” and “Drug-Free Communities evaluation.”

Studies were reviewed if they addressed coalitions as a service delivery system rather than examining the effectiveness of individual prevention interventions. Specifically, studies were included if they assessed coalition adoption across communities or sectors; implementation processes such as fidelity, coordination, training demands, or resource allocation; sustainability and institutionalization over time; structural characteristics (e.g., governance, network functioning, funding stability) that influence coalition performance; and community-level indicators and outputs associated with coalition activity. Studies were excluded if they did not focus on substance misuse prevention coalitions as organizational delivery systems, or if they examined only individual-level outcomes from a single program without assessing coalition structure, functioning, sustainability, or community-level outputs.

In addition to peer-reviewed studies, relevant grey literature was also reviewed to capture federal evaluations, funding reports, and implementation documentation not published in academic journals. Grey literature sources included publicly available reports from SAMHSA, CADCA, Government Accountability Office reports, Congressional Research Service documents, and state-level evaluation summaries. Grey literature was identified through targeted searches of agency websites and citation tracking from peer-reviewed studies. Grey literature was included when it provided formal evaluation findings, funding analyses, or implementation documentation relevant to coalition adoption, implementation, or sustainability. Opinion pieces, advocacy materials, and non-evaluative commentary were not included.

## Results

### Prevention System Performance: Reach and Effectiveness Indicators

The following subsections assess the coalition-based prevention infrastructure through the RE-AIM domains of Reach and Effectiveness. The analysis focuses on population penetration (reach) and national substance use indicators (effectiveness). In this analysis, reach refers to the proportion and representativeness of individuals exposed to prevention programming across the U.S., while effectiveness refers to national population-level changes in substance use patterns, disorder prevalence, overdose mortality, and related societal burden over time. The effectiveness indicators presented in this section provide descriptive, population-level benchmarks and are not intended to establish causal attribution to the federal prevention system or to individual prevention strategies or programs delivered within it. Rather, they contextualize national substance use trends during a period of sustained federal investment in a coalition-based prevention infrastructure and serve as a basis for informing policy decisions regarding return on investment and future prevention funding priorities. The selected indicators align with federal performance goals articulated in the National Drug Control Strategy (ONDCP, 2022), the Surgeon General’s Report on Alcohol, Drugs, and Health (USDHHS, 2016), and SAMHSA’s Strategic Priorities (SAMHSA, 2023a), all of which emphasize national reductions in substance use prevalence, substance use disorder burden, and overdose mortality.

Currently, five primary national data systems measure substance use and related behaviors and serve as de facto evaluation tools for the U.S. prevention system. These include the Behavioral Risk Factor Surveillance System, the NSDUH, the Youth Risk Behavior Survey, the American College Health Association’s National College Health Assessment, and MTF. Given overlapping measures and methodological shifts across surveys, NSDUH data (2002–2019) and MTF data (1992–2019) were examined as standardized indicators of national trends.

The year 2002 was selected as the NSDUH baseline because of a major survey revision implemented that year that limited comparability with prior estimates (SAMHSA, 2020). The year 1992 was selected as the MTF baseline to align with the establishment of SAMHSA and the Substance Use Prevention, Treatment, and Recovery Services Block Grant. The year 2019 was used as the endpoint to avoid confounding effects of the COVID-19 pandemic.

#### Reach

National NSDUH analyses consistently demonstrate declining adolescent participation in both school-based and community-based substance use prevention programs over the past two decades (Lu et al., 2024; Salas-Wright et al., 2019). Salas-Wright et al. (2019) found that school-based prevention participation among adolescents declined from 48.1 to 40.2% between 2002 and 2016, while community-based participation declined from 13.2 to 11.5% during the same period (Salas-Wright et al., 2019). More recent analyses using updated NSDUH measures (2011–2019) similarly found statistically significant declines in both school-based and community-based prevention exposure (Lu et al., 2024), although prevalence estimates differ due to changes in survey operationalization. These findings suggest that exposure to prevention programming has not expanded over time and may have contracted in certain settings. Given that reach represents a prerequisite for system-level effectiveness, limited population penetration may constrain the overall impact of the coalition-based model.

#### Effectiveness

According to NSDUH data, past 30-day alcohol use among young adults aged 18–25 declined from 61% in 2002 to 54% in 2019, while use among adults aged 26 and older remained stable at 54–55% during the same period. Marijuana use increased among both age groups, rising from 17 to 23% among 18–25-year-olds and from 4 to 10% among adults aged 26 and older. In contrast, past 30-day use of illicit drugs other than marijuana declined slightly among 18–25-year-olds, from 8% in 2015 to 6% in 2019, and remained stable at approximately 3% among adults aged 26 and older (SAMHSA, 2020). These trends are presented in Table 2.

**Table 2:** NSDUH past-30-day use (2002–2019)

Substance	Age group	2002	2019	Trend
Alcohol	18–25	61%	54%	Modest decline
	26 +	54%	55%	Stable
Marijuana	18–25	17%	23%	Modest increase
	26 +	4%	10%	Modest increase
Illicit drugs*	18–25	8% (2015)	6%	Slight decline
	26 +	3% (2015)	3%	Stable

*Excludes marijuana

*Stable: − 1.0 to + 1.0 percentage points

*Slight increase/slight decline: 1.1–3.0 percentage points

*Modest increase/modest decline: 3.1–9.9 percentage points

*Sharp increase/sharp decline: ≥ 10.0 percentage points

Monitoring the Future data show that the percentage of 12th graders reporting past 30-day alcohol use declined from 51.3 in 1992 to 29.3% in 2019, while marijuana use increased from 11.9 to 22.3% during the same period. Past 30-day use of illicit drugs other than marijuana increased from 6.3 in 1992 to a peak of 11.3% in 2002 before declining to 5.2% in 2019, returning to below baseline levels. Past 30-day cigarette use declined substantially from 27.8 in 1992 to 5.7% in 2019. However, nicotine vaping reached 25.5% among 12th graders in 2019, offsetting some gains in traditional cigarette reduction (Johnston et al., 2020). These trends are presented in Table 3.

**Table 3:** MTF past 30-day substance use among 12th graders (1992–2019)

Substance	1992	2019	Trend
Alcohol	51.3%	29.3%	Sharp decline
Marijuana	11.9%	22.3%	Sharp increase
Illicit drugs*	6.3%	5.2%	Slight decline
Cigarettes	27.8%	5.7%	Sharp decline
Nicotine vaping	—	25.5%	—

*Excludes marijuana

*Stable: –1.0 to + 1.0 percentage points

*Slight increase/slight decline: 1.1 to 3.0 percentage points

*Modest increase/modest decline: 3.1 to 9.9 percentage points

*Sharp increase/sharp decline: ≥ 10.0 percentage points

In addition to usage rates, two of the most consequential indicators of effectiveness, overdose mortality and economic burden, remain deeply concerning. According to the National Center for Health Statistics, the age-adjusted drug overdose death rate rose from 6.1 deaths per 100,000 in 1999 to 8.9 in 2003, with a peak of 32.6 in 2022 (Hedegaard et al., 2018; Garnet & Miniño, 2024). The economic cost of substance use disorders, encompassing health care, criminal justice, lost productivity, and premature mortality, exceeds $700 billion annually in the U.S. (NIDA, 2020; Fardone et al., 2023).

Although certain substance-specific indicators such as youth cigarette use and prescription opioid misuse have declined over the past decade, overall illicit drug use, substance use disorder prevalence, and overdose mortality have remained persistently high. Since 2021, national estimates indicate that between 46 and 49 million individuals aged 12 or older meet criteria for a past-year substance use disorder annually (SAMHSA, 2024a).

In summary, analysis of national surveillance data over the past two decades indicates that while certain substance-specific indicators have improved, overall population-level reach and sustained reductions in aggregate burden remain limited. Declines in school-based and community-based prevention participation further suggest that exposure to prevention programming has not expanded in proportion to need and may not have been delivered at sufficient scale to influence national population health indicators. Although many evidence-based prevention programs have demonstrated efficacy under controlled conditions (USDHS, 2016), national trends in substance use, overdose mortality, and substance use disorder prevalence suggest that prevention programming has not been implemented with the breadth or consistency required to generate durable, population-level change. Collectively, these patterns raise questions about whether the current coalition-based infrastructure achieves adequate reach and sustained system-level effectiveness as a national prevention strategy.

### Coalition Framework Performance: Adoption, Implementation, and Maintenance

The following subsections assess the coalition-based prevention infrastructure through the RE-AIM domains of Adoption, Implementation, and Maintenance, focusing on organizational uptake and structural capacity (adoption), delivery quality and operational functioning (implementation), and long-term sustainability (maintenance) at the systems level rather than on outcomes of individual prevention programs or curricula.

### Adoption

Adoption within the coalition-based prevention system is highly variable and dependent on local readiness, leadership stability, and contextual factors. Research indicates that coalition uptake and willingness to implement structured prevention approaches are influenced by community capacity, political climate, demographics, and prevailing social norms (Stith et al., 2006; Wang et al., 2024; Zakocs & Edwards, 2006). Because participation is voluntary and not embedded within mandated service systems such as schools or healthcare settings, adoption of evidence-based prevention programs (EBPPs) varies widely across jurisdictions.

Network structure also appears to influence adoption. Smaller, lower-density coalitions may adopt evidence-based practices more effectively than larger, more complex coalitions, suggesting that organizational structure shapes implementation decisions (Fujimoto et al., 2009; Valente et al., 2007). However, adoption of EBPPs is frequently constrained by limited readiness, lack of formal action plans, and insufficient evaluation infrastructure. Arthur et al. (2010) found that while Communities That Care (CTC) coalitions achieved higher fidelity benchmarks than controls, many non-CTC coalitions lacked documented action plans, outcome evaluations, and core structural components necessary for effective coalition work. These findings suggest that adoption of structured, evidence-based prevention strategies is inconsistent and dependent on local organizational capacity rather than systematically ensured across communities.

### Implementation

Implementation challenges are widely documented in the coalition literature. Coalitions require substantial administrative effort to sustain membership, develop partnerships, and conduct regular meetings (Kreuter et al., 2000; Gloppen et al., 2012; Wang et al., 2024; Zakocs & Edwards, 2006). These responsibilities occur alongside programmatic duties such as training, needs assessments, strategy selection, program delivery, and evaluation (Arthur et al., 2010; Durlak & DuPre, 2008; Stith et al., 2006; Valente et al., 2007; Feinberg et al., 2008). Early reviews found only marginal evidence that coalitions improve health or systems outcomes, citing inefficiencies in collaborative mechanisms, unrealistic expectations, and challenges in outcome attribution (Kreuter et al., 2000).

Large-scale demonstrations reinforce these concerns. The Robert Wood Johnson Foundation’s Fighting Back initiative, which provided substantial funding to 14 communities over 5 years, found no evidence that youth or community-focused strategies reduced substance use. Adult-focused strategies were associated with negative outcomes compared to matched controls, and higher-dose or more comprehensive strategies did not yield improved results (Hallfors et al., 2002). These findings suggest that greater intensity of coalition activity does not necessarily translate into superior implementation outcomes.

More broadly, implementation quality is influenced by more than 20 contextual factors, including local capacity, community conditions, readiness, program-community fit, fidelity to evidence-based models, and access to sufficient resources (Durlak & DuPre, 2008; Hutchison & Russell, 2021; Stith et al., 2006). Coalitions are therefore highly sensitive to environmental variability, which complicates consistent, high-quality implementation at scale.

### Maintenance

Sustainability represents one of the most persistent challenges within the coalition framework. Evaluations of CTC coalitions found that 10% of coalitions in Pennsylvania closed immediately after funding ended, and one-third had closed within 4 years (Feinberg et al., 2008). Gloppen et al. (2012) similarly found that although most coalitions remained active shortly after external support ended, many experienced funding declines, loss of paid staff, and reductions in implemented EBPPs. Only a subset maintained documented action plans over time.

These findings indicate that coalition maintenance is closely tied to continued external funding and technical assistance. Without sustained financial support, many coalitions reduce programming intensity or cease operations entirely. The model therefore requires ongoing federal investment not only to initiate but also to maintain infrastructure. Such funding dependence raises concerns regarding long-term sustainability and return on investment, particularly when population-level outcomes remain inconsistent.

Overall, evidence from more than three decades of research raises concerns about the adoption, implementation quality, and sustainability of the community coalition model as a national strategy for delivering prevention services in the USA. While individual evidence-based prevention programs have demonstrated effectiveness in preventing substance misuse and delaying initiation (USDHS, 2016), the coalition model itself has been associated with structural, contextual, and implementation challenges that render it resource-intensive, unevenly adopted, difficult to implement with fidelity, and inconsistently sustained. Notably, the variability observed across coalitions appears not to reflect isolated implementation failures, but recurring structural features of the model. Because the approach relies on voluntary participation, multi-sector coordination, leadership stability, and sustained local capacity, high-quality implementation is inherently difficult to standardize at scale. Collectively, findings across adoption, implementation, and maintenance domains suggest that although coalitions can mobilize local stakeholders, the model has limited capacity to generate durable, population-level impact at scale. These limitations highlight the need to reconsider how federal prevention resources are allocated and to explore alternative approaches that may offer greater scalability, efficiency, and long-term sustainability.

## Alternative Funding Priorities for Prevention

To address the limitations of the coalition-based system, this paper proposes five funding priorities for restructuring federal prevention efforts. These new priorities include the following: (1) Training and education sustainability, (2) Implementation setting efficiency, (3) Screening and early intervention, (4) Decommodification of prevention strategies and programs, and (5) Technological innovation. Collectively, these priorities provide a pathway to expand the use of EBPPs, strengthen evaluation capacity, and reduce costs while improving return on investment and enhancing the long-term sustainability of the prevention system. The proposed priorities are intentionally structured to address the recurring structural constraints identified in the AIM analysis. Rather than relying on voluntary multi-sector collaboration, sustained local political alignment, and fluctuating external grant support, these alternatives embed prevention within existing institutional infrastructures such as professional education systems, licensure requirements, mandated school programming, centralized procurement mechanisms, and scalable digital platforms. By shifting prevention from a discretionary, coalition-dependent model to a standardized institutional function, the proposed framework seeks to enhance scalability, consistency, and long-term sustainability while creating a clearer pathway for influencing national substance use indicators.

### Training and Education Sustainability

The goal of this priority is to integrate prevention training into existing education and licensure structures so that prevention knowledge is embedded sustainably across disciplines such as social work, public health, and education. By incorporating training for teachers, social workers, and other primary implementers into professional programs, reliance on external organizations such as CADCA could be reduced, thereby improving the economic efficiency of limited funding and increasing the accessibility and consistency of EBPP delivery across communities. Integrating prevention science into teacher education and professional licensure could also create a cost-free, sustainable programming pipeline. Embedding training in EBPPs, such as the Good Behavior Game or Botvin LifeSkills Training, into certification requirements for teachers would further ensure consistent preparation across schools and reduce dependence on external training and technical assistance. Given documented gaps in addictions education across academic training programs for prevention-aligned professions including social work, nursing, psychology, and education, this priority directly addresses a structural limitation of the current service delivery system (Minnick, 2021; Park & Minnick, 2023; Rigg et al., 2025; U.S. Department of Health and Human Services [USDHS], 2016).

### Implementation Setting Efficiency

The goal of this priority is to ensure that prevention programming is systematically integrated into settings where it can have the greatest reach and impact, beginning with schools. With the objective of mandating EBPP delivery through state or federal policy, effective prevention programming could become a standard educational practice. As noted in the 2016 Surgeon General’s Report, only 8–10% of school administrators reported using EBPPs, and a mandate tied to block grant and educational funding streams could directly close this gap, expand reach, and ensure consistent implementation nationwide (USDHS, 2016). In addition to schools, funding could also directly target other high-engagement youth settings such as sports or theatre programs and after-school activities. These venues already engage large numbers of youth in supportive settings, enabling prevention programming to be delivered with fewer external resources and potentially generating a stronger return on investment than the current coalition-based model, especially if aligned with proposed education and training enhancements.

### Screening and Early Intervention

The goal of this priority is to normalize early detection of substance use risk by embedding routine screening into schools and healthcare systems in the same routine manner that blood pressure, cholesterol, or diabetes screenings are conducted with adult populations. When paired with non-punitive, evidence-based interventions, this approach reframes substance use as a universal public health concern (one which all youth are exposed to) and as an issue warranting systematic monitoring and early action, rather than as a problem limited to specific groups or managed primarily through consequence and interdiction activities. As with other public health screenings, widespread implementation would support earlier identification, enable timely intervention, decrease stigma, and contribute to stronger long-term health outcomes at the population level. Integrating screening and early intervention within a school-service framework such as Multi-Tiered Systems of Support (MTSS) may also further enhance feasibility and impact. MTSS prioritizes proactive screening and tiered intervention delivery aligned with universal, selective, and indicated prevention categories (Institute of Medicine, 1994), while coordinating academic and behavioral supports within existing school infrastructure. Embedding substance misuse screening within this structure would allow early identification and tiered response to be delivered systematically, strengthening prevention practices and potentially improving downstream treatment engagement and outcomes (Nitz et al., 2023). It may also reduce strain on under-resourced and overburdened school-service systems by integrating prevention into existing service delivery pathways rather than adding parallel structures.

### Decommodification of Prevention Strategies and Programs

The goal of this priority is to reduce financial and structural barriers to EBPP adoption by providing schools, communities, and providers with direct access to evidence-based prevention programs at no cost. A centralized, open-access EBPP repository could replace the current patchwork of costly acquisition processes and reduce dependence on proprietary training and registry systems associated with professional organizations or state agencies such as Blueprints or the New York State Registry of Evidence-Based Programs.

As one operational example, the federal government could reinvest in a national prevention clearinghouse modeled structurally on the former National Registry of Evidence-Based Programs and Practices, but redesigned to function as a curated repository rather than solely as a registry. Under this model, federal agencies could negotiate licensing agreements or purchase program rights where appropriate to make selected EBPPs freely available to schools and providers. Federal funding could also support the development of new prevention programs, expansion of the repository catalog, and ongoing scientific review of existing programs and new submissions. Oversight and site maintenance could be administered through internal federal management or through an independent expert review panel, similar to existing state-level models such as New York State’s Evidence-Based Prevention Program Review Panel (NYS-OASAS, 2022).

The review body would establish clear program standards and evidence criteria to ensure transparency, scientific rigor, and procedural consistency. For each included program, standardized fidelity guidance and implementation supports could be developed to promote quality delivery in real-world settings, while oversight of strategy selection and implementation would remain under the purview of individual states, consistent with the current federal structure. This approach would preserve intellectual property protections through negotiated agreements while reducing financial barriers for communities and maintaining quality oversight. Developer-led training and technical assistance could continue, particularly for more complex interventions, but access to core program materials would no longer be restricted by cost. By centralizing procurement and review functions, this resource would allow technical assistance providers to focus more directly on implementation quality, fidelity monitoring, and evaluation alignment rather than on sustaining fragmented acquisition systems. Regardless of the specific governance structure adopted, this model has the potential to substantially reduce community costs, expand EBPP utilization, and strengthen the national prevention evidence base in a systematic and sustainable manner.

### Digital Applications and Wearables

The goal of the final priority is to expand prevention access through the use of digital tools and emerging technologies. Mobile applications, telehealth platforms, and wearable devices can deliver prevention programming in scalable, personalized ways, especially in rural or underserved areas (Carreiro et al., 2018; Kazemi et al., 2017; Lin et al., 2019). Advances in artificial intelligence and biometric monitoring could also provide real-time feedback, track behaviors, and trigger timely interventions. Prioritizing these technologies within the prevention ecosystem would establish new standards for individualized, technology-enabled prevention, and greatly expand access to quality and impactful programming.

Table 4 provides a detailed comparison of the proposed priorities with the current coalition-based system.

**Table 4:** Proposed vs. current prevention system

System component	Proposed system	Current system
Training and education sustainability	Prevention science is integrated into relevant degree programs (e.g., teaching, education, social work, counseling, public health) and required for practice and licensure to work in relevant practice settings	Workforce is commonly trained on the job; most states lack standardized pre-service education or licensure requirements. Prevention training is not mandated or routinely offered in relevant higher education courses, degrees, or contexts
Strategy implementation settings	Mandates universal prevention curricula in school systems and other scalable, prevention-centered environments	Prevention is inconsistently integrated across schools and communities; implementation is heavily dependent on local relationships, district and community leadership, and funding variability
Screening and early intervention	Embeds early and routine screening within standard care protocols and promotes broad, equitable access to supportive interventions	Screening for substance use is rare or inconsistent; responses are often punitive rather than supportive and administered by untrained professionals
National EBPP repository	Establishes a centralized, open-access repository of evidence-based prevention programs and tools, regularly updated and curated for immediate public use at no cost	No unified or accessible registry; program selection is ad hoc and cost-prohibitive for most communities. Existing registries (e.g., EBPP lists) are difficult to navigate and keep up to date
Digital apps and wearables	Prioritizes development and adoption of validated digital interventions and wearable technologies to support personalized, real-time prevention that is universally accessible	Relies primarily on in-person programming or adoption of public policies; limited use of technology; many utilized interventions are outdated, lack evidence of effectiveness, or are too resource intensive for sustainability

## Discussion and Implications

The RE-AIM analysis highlights stark contrasts between the coalition-based model and proposed alternative prevention framework. While coalitions have demonstrated an ability to mobilize stakeholders and promote local ownership, these strengths come at a high cost. RE-AIM analysis findings suggest that coalition reach is generally uneven, their effectiveness inconsistent, and their long-term sustainability weak, largely because success depends on variable local leadership, substantial external funding, favorable environmental conditions, and extensive ongoing technical assistance. In short, coalitions operate more as relational or promotional mechanisms than as dependable drivers of prevention outcomes, offering a poor return on investment for a system that costs over $665 million annually to sustain.

By contrast, the proposed framework embeds evidence-based prevention within existing infrastructure including schools, healthcare systems, and digital platforms, which are already systematically integrated into nearly every community. This approach also improves reach by making prevention a routine element of core community institutions rather than an external, grant-dependent activity. Effectiveness is further enhanced through the systematic use of free, evidence-based programs and universal screenings, while workforce development and integration into licensure requirements ensure a sustainable pipeline of trained practitioners. Digital applications and wearables extend reach even further by enabling personalized, scalable prevention that is not constrained by geography or coalition capacity, which can also address equity concerns in areas where a lack of volunteers or other local environmental factors can reduce accessibility to prevention programming.

From a policy perspective, the coalition model currently directs substantial federal and state resources toward maintaining administrative structures rather than delivering EBPPs at scale. A modernized framework reallocates those funds to universal infrastructures, linking block-grant allocations to EBPP mandates, professional training, and the creation of a national repository of free, evidence-based programs. This shift prioritizes efficiency, accountability, and equity.

In sum, the coalition model is politically attractive but operationally ineffective and inefficient, while the proposed model offers a theoretically more scalable, sustainable, and cost-effective strategy for achieving population-level reductions in substance misuse. Shifting federal prevention priorities toward this approach would modernize infrastructure, maximize return on investment, and align substance misuse prevention with broader, evidence-based public health practice.

## Limitations

Several limitations should be noted in interpreting the findings of this exploratory analysis. First, the indicators used to assess prevention system performance, including national surveys of substance use, overdose mortality data, and economic cost estimates, are imperfect and function as rough proxies rather than precise measures. Self-reported survey data are subject to reporting biases, while mortality and cost estimates capture multiple interacting factors beyond the scope of prevention programming. The ongoing opioid epidemic further complicates evaluation. The rapid emergence of synthetic opioids, particularly fentanyl, has driven sharp increases in mortality over the past decade, making it difficult to disentangle the specific contribution of prevention strategies and coalitions from broader shifts in the drug supply. Evolving drug laws and changing social norms have also added additional complexity. The legalization and decriminalization of marijuana, shifting enforcement priorities, and growing acceptance of cannabis use have also altered both prevalence rates and perceptions of risk, which likely affect substance use trends independent of prevention programming. Finally, as noted in the literature, assessing population-level impacts of prevention is inherently difficult. Prevention outcomes often emerge gradually, may be context-specific, and can be obscured by broader social, economic, and cultural factors. Establishing causal links between specific prevention strategies and national-level outcomes remains challenging, underscoring the need for more rigorous, longitudinal, and system-level evaluations.

## Conclusion

As shown in the findings and discussion, modernizing the U.S. prevention system requires a deliberate transition from a coalition-based framework to one embedded in core American institutions. Future research should explore strategies to phase out reliance on coalitions while building prevention capacity into professional education, licensure, and universal service settings. Evaluation must extend beyond short-term outputs to include system-level outcomes such as scalability, fidelity, sustainability, and return on investment. Rigorous studies of digital tools, the development of new EBPPs, the creation of a national prevention repository, and advanced models for integrating screening protocols will be critical to guiding this shift. Collectively, these steps can establish an evidence-driven, cost-efficient prevention infrastructure capable of delivering consistent public health impact at scale.

## References

[CR1] Arthur, M. W., Hawkins, J. D., Brown, E. C., Briney, J. S., & Oesterle, S. (2010). Implementation of the communities that care prevention system by coalitions in the community youth development study. *Journal of Community Psychology,**38*(2), 245–258. 10.1002/jcop.20362 22199409 10.1002/jcop.20362 PMC3244354

[CR2] Butterfoss, F. D., & Kegler, M. C. (2002). Toward a comprehensive understanding of community coalitions. *Emerging theories in health promotion practice and research*, 157–193.

[CR3] Carreiro, S., Chai, P. R., Carey, J., & Boyer, E. W. (2018). mHealth for the detection and intervention in adolescent and young adult substance use disorder. *Current Addiction Reports,**5*(1), 110–119. 10.1007/s40429-018-0192-0 30148037 10.1007/s40429-018-0192-0 PMC6107303

[CR4] Centers for Disease Control and Prevention. (2024). *Youth risk behavior survey: Data summary & trends report, 2003–2023.* U.S. Department of Health & Human Services.

[CR5] Centers for Disease Control and Prevention. (2025) *Drug-Free Communities Coalitions*. https://www.cdc.gov/overdose-prevention/php/drug-free-communities/coalitions.html

[CR6] Community Anti-Drug Coalition of America [CADCA]. (n.d.). *About CADCA.* Community Anti-Drug Coalitions of America. https://www.cadca.org/about

[CR7] Community Anti-Drug Coalitions of America. (2018). *National Coalition Institute primer series: Primer handbook*. Community Anti-Drug Coalitions of America*.* https://www.cadca.org/wp-content/uploads/2019/02/handbookcompressed.pdf

[CR8] Community Anti-Drug Coalitions of America. (2023). *Schedule of expenditures of federal awards, year ended December 31, 2023* [Audited financial statement]. Retrieved from https://www.cadca.org/about/financials

[CR9] Duff, J. (2020). *The substance abuse and mental health services administration (SAMHSA): Overview and funding* (CRS Report No. R46426). Congressional Research Service. https://www.congress.gov/crs-product/R46426

[CR10] Durlak, J. A., & DuPre, E. P. (2008). Implementation matters: A review of research on the influence of implementation on program outcomes and the factors affecting implementation. *American Journal of Community Psychology,**41*(3–4), 327–350. 10.1007/s10464-008-9165-0 18322790 10.1007/s10464-008-9165-0

[CR11] Elicit. (2025). *Elicit: The AI Research Assistant.* https://elicit.com

[CR12] Fardone, E., Montoya, I. D., Schackman, B. R., & McCollister, K. E. (2023). Economic benefits of substance use disorder treatment: A systematic literature review of economic evaluation studies from 2003 to 2021. *Journal of Substance Use & Addiction Treatment,**152*, Article 209084. 10.1016/j.josat.2023.209084 37302488 10.1016/j.josat.2023.209084 PMC10530001

[CR13] Feinberg, M. E., Bontempo, D. E., & Greenberg, M. T. (2008). Predictors and level of sustainability of community prevention coalitions. *American Journal of Preventive Medicine,**34*(6), 495–501. 10.1016/j.amepre.2008.01.030 18471585 10.1016/j.amepre.2008.01.030

[CR14] Fujimoto, K., Valente, T. W., & Pentz, M. A. (2009). Network structural influences on the adoption of evidence-based prevention in communities. *Journal of Community Psychology,**37*(7), 830–845. 10.1002/jcop.20333 28883667 10.1002/jcop.20333 PMC5584875

[CR15] Gaglio, B., Shoup, J. A., & Glasgow, R. E. (2013). The RE-AIM framework: A systematic review of use over time. *American Journal of Public Health,**103*(6), e38–e46. 10.2105/AJPH.2013.301299 23597377 10.2105/AJPH.2013.301299 PMC3698732

[CR16] Glasgow, R. E., Harden, S. M., Gaglio, B., Rabin, B., Smith, M. L., Porter, G. C., Ory, M. G., & Estabrooks, P. A. (2019). RE-AIM planning and evaluation framework: Adapting to new science and practice with a 20-year review. *Frontiers in Public Health,**7*, Article 64. 10.3389/fpubh.2019.00064 30984733 10.3389/fpubh.2019.00064 PMC6450067

[CR17] Glasgow, R. E., Vogt, T. M., & Boles, S. M. (1999). Evaluating the public health impact of health promotion interventions: The RE-AIM framework. *American Journal of Public Health,**89*(9), 1322–1327. 10.2105/AJPH.89.9.1322 10474547 10.2105/ajph.89.9.1322 PMC1508772

[CR18] Gloppen, K. M., Arthur, M. W., Hawkins, J. D., & Shapiro, V. B. (2012). Sustainability of the communities that care prevention system by coalitions participating in the community youth development study. *Journal of Adolescent Health,**51*(3), 259–264. 10.1016/j.jadohealth.2011.12.018 10.1016/j.jadohealth.2011.12.018 PMC3428591 22921136

[CR19] Hallfors, D., Cho, H., Livert, D., & Kadushin, C. (2002). Fighting back against substance abuse: Are community coalitions winning? *American Journal of Preventive Medicine,**23*(4), 237–245. 10.1016/S0749-3797(02)00511-1 12406477 10.1016/s0749-3797(02)00511-1

[CR20] Hedegaard, H., Miniño, A. M., & Warner, M. (2018). *Drug overdose deaths in the United States, 1999–2017* (NCHS Data Brief, No. 329). National Center for Health Statistics. https://www.cdc.gov/nchs/products/databriefs/db329.htm 30500323

[CR21] HigherGov. (2023, November 9). *2022–0255-AMENDMENT-1 (subcontract under SAMHSA SPTAC prime award 75S20322D00008–75S20322F42001: Carnevale associates LLC).* Retrieved October 2, 2025, from https://www.highergov.com/subcontract/75S20322D00008-75S20322F42001-2022-0255-AMENDMENT-1/

[CR22] Hutchison, M., & Russell, B. S. (2021). Community coalition efforts to prevent adolescent substance use: A systematic review. *Journal of Drug Education,**50*(1–2), 3–30. 10.1177/00472379211016384 34078126 10.1177/00472379211016384

[CR23] Institute of Medicine (US) Committee on Prevention of Mental Disorders, Mrazek, P. J., & Haggerty, R. J. (Eds.). (1994). *Reducing risks for mental disorders: Frontiers for preventive intervention research*. National Academies Press (US). 10.17226/2139 25144015

[CR24] Jilcott, S., Ammerman, A., Sommers, J., & Glasgow, R. E. (2007). Applying the RE-AIM framework to assess the public health impact of policy change. *Annals of Behavioral Medicine,**34*(2), 105–114. 10.1007/BF02872666 17927550 10.1007/BF02872666

[CR25] Johnston, L. D., Miech, R. A., O’Malley, P. M., Bachman, J. G., Schulenberg, J. E., & Patrick, M. E. (2020). *Monitoring the future national survey results on drug use, 1975–2019: Overview, key findings on adolescent drug use.* Institute for Social Research, University of Michigan. https://monitoringthefuture.org/wp-content/uploads/2022/08/mtf-overview2019.pdf

[CR26] Kane, C. (2021). *Office of National Drug Control Policy and its role in federal drug control* (CRS Report No. R46889). Congressional Research Service. https://www.congress.gov/crs-product/R46889

[CR27] Kazemi, D. M., Borsari, B., Levine, M. J., Li, S., Lamberson, K. A., & Matta, L. A. (2017). A systematic review of the mhealth interventions to prevent alcohol and substance abuse. *Journal of Health Communication,**22*(5), 413–432. 10.1080/10810730.2017.1303556 28394729 10.1080/10810730.2017.1303556 PMC5616128

[CR28] Kessler, R. S., Purcell, E. P., Glasgow, R. E., Klesges, L. M., Benkeser, R. M., & Peek, C. J. (2012). What does it mean to “employ” the RE-AIM model? *Evaluation & the Health Professions,**36*(1), 44–66. 10.1177/0163278712446066 22615498 10.1177/0163278712446066

[CR29] Kreuter, M. W., Lezin, N. A., & Young, L. A. (2000). Evaluating community-based collaborative mechanisms: Implications for practitioners. *Health Promotion Practice,**1*(1), 49–63.

[CR30] Lau, O., & Golder, S. (2025). Comparison of elicit AI and traditional literature searching in evidence syntheses using four case studies. *Cochrane Evidence Synthesis and Methods,**3*(6), Article e70050. 10.1002/cesm.70050 41035533 10.1002/cesm.70050 PMC12483133

[CR31] Lin, L. A., Casteel, D., Shigekawa, E., Weyrich, M. S., Roby, D. H., & McMenamin, S. B. (2019). Telemedicine-delivered treatment interventions for substance use disorders: A systematic review. *Journal of Substance Abuse Treatment,**101*, 38–49. 10.1016/j.jsat.2019.03.007 31006553 10.1016/j.jsat.2019.03.007

[CR32] Lu, Y., Salas-Wright, C. P., Vaughn, M. G., & Todic, J. (2024). Trends in adolescent participation in school- and community-based substance use prevention programs in the United States, 2011–2019. *Preventive Medicine,**185*, Article 108050. 10.1016/j.ypmed.2024.108050 38906276 10.1016/j.ypmed.2024.108050

[CR33] Minnick, D. (2021). Examining substance use education in social work: A survey of MSW program leaders. *Journal of Social Work Education,**57*(2), 299–315.

[CR34] National Association of State Alcohol and Drug Abuse Directors. (2023). *FY 2023 appropriations for substance use prevention, treatment, and recovery services*. NASADAD. https://nasadad.org/wp-content/uploads/2023/02/FY-2023-Appropriations_Final.pdf

[CR35] National Association of State Alcohol and Drug Abuse Directors. (2025). *Final FY 2025 appropriations chart*. https://nasadad.org/wp-content/uploads/2025/05/Final-FY-25-Appropriations-Chart.pdf

[CR36] Garnett, M. F., & Miniño, A. M. (2024). Drug overdose deaths in the United States, 2003–2023 (NCHS Data Brief No. 522). National Center for Health Statistics. 10.15620/cdc/170565

[CR37] National Institute on Drug Abuse. (2020). *Trends & statistics.* National Institutes of Health. https://nida.nih.gov/publications/research-reports/trends-statistics

[CR38] New York State Office of Addiction Services and Supports. (2022). *Prevention guidelines*. https://oasas.ny.gov/system/files/documents/2023/03/2022_prevention_guideines.pdf

[CR39] Nitz, J., Brack, F., Hertel, S., Krull, J., Stephan, H., Hennemann, T., & Hanisch, C. (2023). Multi-tiered systems of support with focus on behavioral modification in elementary schools: A systematic review. *Heliyon,**9*(6), Article e17506. 10.1016/j.heliyon.2023.e17506 37408895 10.1016/j.heliyon.2023.e17506 PMC10319208

[CR40] Office of National Drug Control Policy. (2022). *National drug control strategy*. Executive Office of the President. https://bidenwhitehouse.archives.gov/wp-content/uploads/2022/04/National-Drug-Control-2022Strategy.pdf

[CR41] Park, D., & Minnick, D. (2023). Examining entry-level social workers’ substance use prevention capacities in a Northeastern State: A field perspective. *Journal of Social Work Practice in the Addictions,**23*(4), 313–324. 10.1080/1533256X.2023.2235596

[CR42] Prevention Technology Transfer Center (PTTC) Network. (n.d.). *About the PTTC network.* Substance Abuse and Mental Health Services Administration. https://pttcnetwork.org/about-the-pttc-network/

[CR43] Rigg, K. K., Proctor, S. L., Rigg, L. K., Faber, K. B., & Pusey-Rigg, L. M. (2025). Why all behavioral health providers need addiction training. *The Journal of Behavioral Health Services & Research*, 1–9. 10.1007/s11414-025-09977-9 41145759

[CR44] Salas-Wright, C. P., Vaughn, M. G., Ugalde, J., & Todic, J. (2019). Trends in substance use prevention program participation among adolescents in the U.S. *Journal of Adolescent Health,**65*(3), 426–429. 10.1016/j.jadohealth.2019.03.021 10.1016/j.jadohealth.2019.04.010 PMC6708775 31277992

[CR45] Stith, S., Pruitt, I., Dees, J., Fronce, M., Green, N., Som, A., & Linkh, D. (2006). Implementing community-based prevention programming: A review of the literature. *Journal of Primary Prevention,**27*(6), 599–617. 10.1007/s10935-006-0062-8 17051431 10.1007/s10935-006-0062-8

[CR46] Substance Abuse and Mental Health Services Administration. (2024a). *2023 National Survey on Drug Use and Health: Detailed tables and results* (HHS Publication No. PEP24-07-01-006, NSDUH Series H-59). Center for Behavioral Health Statistics and Quality, Substance Abuse and Mental Health Services Administration. https://www.samhsa.gov/data/sites/default/files/reports/rpt47095/National%20Report/National%20Report/2023-nsduh-annual-national.pdf

[CR47] Substance Abuse and Mental Health Services Administration. (2024d). *Prevention Technology Transfer Centers (PTTC) (SP-24–002)* [Grant announcement]. https://www.samhsa.gov/grants/grant-announcements/sp-24-002

[CR48] Substance Abuse and Mental Health Services Administration. (2024c). *About SPTAC. U.S. Department of Health & Human Services*. https://www.samhsa.gov/technical-assistance/sptac/about

[CR49] Substance Abuse and Mental Health Services Administration. (2024b). *Strategic prevention framework-Partnerships for success (SPF-PFS) grant program.* U.S. Department of Health & Human Services. https://www.samhsa.gov/substance-use/prevention/strategic-prevention-framework-partnerships-success.

[CR50] Substance Abuse and Mental Health Services Administration. (2019). *Strategic prevention framework* (HHS Publication No. PEP19–01). U.S. Department of Health & Human Services. https://library.samhsa.gov/sites/default/files/strategic-prevention-framework-pep19-01.pdf

[CR51] Substance Abuse and Mental Health Services Administration. (2020). *Key substance use and mental health indicators in the United States: Results from the 2019 National Survey on Drug Use and Health* (HHS Publication No. PEP20–07–01–001, NSDUH Series H-55). Center for behavioral health statistics and quality, substance abuse and mental health services administration. https://library.samhsa.gov/product/results-2019-national-survey-drug-use-and-health-nsduh-key-substance-use-and-mental-health

[CR52] Substance Abuse and Mental Health Services Administration. (2023a). *Strategic priorities*. U.S. Department of Health and Human Services. https://www.samhsa.gov/about/strategic-priorities

[CR53] Substance Abuse and Mental Health Services Administration. (2023b). *FY 2023 Congressional budget justification.* U.S. Department of Health & Human Services. https://www.samhsa.gov/about-us/budget

[CR54] U.S. Department of Health and Human Services. (2016). *Facing addiction in America: The Surgeon General’s report on alcohol, drugs, and health.* https://www.hhs.gov/sites/default/files/facing-addiction-in-america-surgeon-generals-report.pdf 28252892

[CR55] United States Congress. (1992). *ADAMHA Reorganization Act of 1992,* Pub. L. No. 102–321, 106 Stat. 323.

[CR56] United States Congress. (1997). *Drug-Free Communities Act of 1997,* Pub. L. No. 105–20, 111 Stat. 224.

[CR57] USAspending.gov. (n.d.). *Search results: CADCA awards* [Data search]. U.S. Department of the Treasury. Retrieved October 2, 2025, from https://www.usaspending.gov/search?hash=b715de2f803058ae4cdabebd164d2586

[CR58] Valente, T. W., Chou, C.-P., & Pentz, M. A. (2007). Community coalitions as a system: Effects of network change on adoption of evidence-based substance abuse prevention. *American Journal of Public Health,**97*(5), 880–886. 10.2105/AJPH.2005.063644 17329667 10.2105/AJPH.2005.063644 PMC1854884

[CR59] Wang, Y., Orwenyo, E. K., Gilmore Powell, K., Peterson, N. A., Wang, Y., Borys, S., & Hallcom, D. K. (2024). Dimensions of community context that affect coalition effectiveness: Development of an instrument. *Journal of Social Work Practice in the Addictions,**25*, 183–198. 10.1080/1533256X.2024.2414984

[CR60] Zakocs, R. C., & Edwards, E. M. (2006). What explains community coalition effectiveness? A review of the literature. *American Journal of Preventive Medicine,**30*(4), 351–361. 10.1016/j.amepre.2005.12.004 16530624 10.1016/j.amepre.2005.12.004

